# The use of caspase inhibitors in pulsed-field gel electrophoresis may improve the estimation of radiation-induced DNA repair and apoptosis

**DOI:** 10.1186/1748-717X-6-6

**Published:** 2011-01-15

**Authors:** Josep Balart, Gemma Pueyo, Lara I de Llobet, Marta Baro, Xavi Sole, Susanna Marin, Oriol Casanovas, Ricard Mesia, Gabriel Capella

**Affiliations:** 1 Translational Research Laboratory - IDIBELL, Institut Català d'Oncologia, L'Hospitalet de Llobregat, Spain; 2 Biostatistics & Bioinformatics Unit, Department of Epidemiology and Cancer Registry, Institut Català d'Oncologia, L'Hospitalet de Llobregat, Spain; 3 Department of Radiation Oncology, Institut Català d'Oncologia, L'Hospitalet de Llobregat, Spain; 4 Department of Medical Oncology, Institut Català d'Oncologia, L'Hospitalet de Llobregat, Spain; 5 Department of Radiation Oncology, Hospital de la Santa Creu i Sant Pau, Barcelona, Spain

## Abstract

**Background:**

Radiation-induced DNA double-strand break (DSB) repair can be tested by using pulsed-field gel electrophoresis (PFGE) in agarose-encapsulated cells. However, previous studies have reported that this assay is impaired by the spontaneous DNA breakage in this medium. We investigated the mechanisms of this fragmentation with the principal aim of eliminating it in order to improve the estimation of radiation-induced DNA repair.

**Methods:**

Samples from cancer cell cultures or xenografted tumours were encapsulated in agarose plugs. The cell plugs were then irradiated, incubated to allow them to repair, and evaluated by PFGE, caspase-3, and histone H2AX activation (γH2AX). In addition, apoptosis inhibition was evaluated through chemical caspase inhibitors.

**Results:**

We confirmed that spontaneous DNA fragmentation was associated with the process of encapsulation, regardless of whether cells were irradiated or not. This DNA fragmentation was also correlated to apoptosis activation in a fraction of the cells encapsulated in agarose, while non-apoptotic cell fraction could rejoin DNA fragments as was measured by γH2AX decrease and PFGE data. We were able to eliminate interference of apoptosis by applying specific caspase inhibitors, and improve the estimation of DNA repair, and apoptosis itself.

**Conclusions:**

The estimation of radiation-induced DNA repair by PFGE may be improved by the use of apoptosis inhibitors. The ability to simultaneously determine DNA repair and apoptosis, which are involved in cell fate, provides new insights for using the PFGE methodology as functional assay.

## Background

The use of pulsed-field gel electrophoresis (PFGE) is widespread in the evaluation of DNA fragmentation caused by double-strand breaks (DSBs) following ionizing radiation [1-4]. The DNA-DSBs may result in the formation of small (often acentric) chromosomal fragments. Following this initial damage, cells activate DNA repair mechanisms to prevent catastrophic mitosis and cell death due to the loss of acentric DNA fragments [5]. The principle of PFGE methodology is that the release of DNA from cells correlates adequately with the intensity of DNA fragmentation [6]. The estimation of DNA repair by PFGE is based on the diminution of DNA released from cells as the length of the DNA fragments increases through the process of rejoining. Thus, a decrease in the ratio of DNA extracted from the cells over a period of time can be used as an evaluation of DNA repair [7].

In the PFGE technique, cells are encapsulated in agarose to form cell-plugs, thus preventing physic damage of the cells while facilitating their manipulation and placement into agarose gels where electrophoresis will take place. Usually in laboratory routine, cells are encapsulated after a period of repair which is allowed to occur in physiological conditions such as either cell cultures or xenografts. Thus, extraction ratios depend exclusively on induced and repaired DNA damage. While the desired strategy is to encapsulate cells after the period of repair has finalized, in a clinical setting, where the availability of cells is limited by the small size of tumour biopsies, it is crucial to concentrate cells in agarose plugs-- to obtain enough cell number for PFGE analysis -- before irradiation [8]. However, this tactic means that DNA breakage and repair occurs in a non-physiological environment. Whitaker and McMillan reported in 1992 that encapsulating cells before irradiation impair the estimation of DNA repair due to the interference of spontaneous DNA fragmentation [8]. This pioneering observation has been confirmed by other studies [9] leading to the belief that results from these sorts of studies (conditions) are not robust enough to properly estimate DNA repair. Nevertheless, the underlying mechanisms affecting cells embedded in agarose during DNA repair in PFGE methodology are still not well understood.

To better understand spontaneous DNA breakage--whether cells are irradiated or not--while cells are encapsulated, we decided to examine PFGE outcomes in cell-plugs over a period of incubation. Our main findings were that 1) incubation of agarose-encapsulated cells induces DNA fragmentation, 2) spontaneous DNA breakage is caused by apoptosis (which can be inhibited by caspase inhibitors), and 3) reducing the interference of spontaneous breakage improves our ability to estimate DNA repair and to determine apoptosis intensity.

## Methods

### Cell lines and tumour xenografts

The tumour cells used in this study were human squamous carcinoma A431 from the American Type Cell Collection (LGC Promochem, Barcelona, Spain), and human pancreatic carcinoma NP18 cell line. NP18 cell line was perpetuated in our laboratory as cell culture and xenografts in nude mice [10,11]. Six-to-eight-week old male athymic Swiss nu/nu mice (Harlan, Gannat, France) were housed at our facilities (Association for Assessment and Accreditation of Laboratory Animal Care accreditation number 1155). Tumours were generated through subcutaneous cell injection of one million NP18 cells into the flank of each mouse. All experimental procedures were approved in accordance with our own institutional guidelines for animal care and ethics. When tumours reached 10 mm in size, they were excised, minced and incubated for 90 min in Dubelcco's Modified Eagle Medium (DMEM) (pH 7.4) containing collagenase type IV (Sigma Aldrich Chemical, Saint Louis, MO, US), and pronase E (Sigma). Then, cell suspensions were incubated for 30 min in trypsin (BioWhittaker, Verviers, Belgium), all at 37°C in gentle agitation. Cell suspensions were finally passed through a 70 μm nylon cell strainer (BD Falcon, Bedford, MA, US).

### DNA double-strand breaks assay for estimation of rejoining using PFGE

Cell pellets obtained following monolayer harvesting or solid tumour processing were mixed with 1% agarose type VII (Sigma). Homogenous aliquots were pipetted into 80 μL plug moulds (Bio-Rad, Hercules, CA, US) to form cell-plugs, adjusting the number of cells per plug to 100,000 cells. Because the volume of the pellets from tumours was slightly smaller than pellets from cell culture, we decided to form cell-plugs using the entire pellet obtained after tumour disaggregation. Cell-plugs were chilled at 4°C for 20 minutes, transferred to DMEM-filled 500 μL tubes, placed on ice, and irradiated at a dose rate of 2.7 Gy/min (6 MV X-rays) up to 45 Gy, a common dose level in PFGE methodology [12-14]. Following irradiation, the medium was replaced with pre-incubated medium at 37°C in a 5% CO_2 _incubator. DNA Repair was stopped by putting the cell-plugs on ice at 0, 0.5, 1, 2 or 4 h after irradiation. Sham unirradiated cell-plugs were managed in parallel with irradiated cell-plugs.

Before electrophoresis, cell-plugs were transferred into ice-cold lysis buffer (pH 7.4) containing 2% sodium lauroyl-sarkosine (Fluka Chemie, Buchs, Switzerland), and 0.5 mg/mL proteinase-K (Sigma) in 0.5 M ethylenediaminetetraacetic acid (EDTA) (Sigma). Lysis was performed on ice for 1 h and then at 37°C for 24 h. At this point, due to the fragility of agarose plugs, the corners tended to break off easily. Therefore, to ensure that the same number of cells (DNA) per lane was loaded into a gel, we cut a section of the better preserved central area. We created a specialized plug cutting device to obtain a section measuring exactly 40 μL. Gels were made of 1% low-melting point agarose, type IX (Sigma), in 0.5 × Tris-Borate-EDTA buffer (TBE) (Sigma), pH 8.4. DNA fragments were resolved by PFGE (CHEF-DR-III, Bio-Rad). Electric field strength was 1.6 V/cm with a switching pulse of 3,600 seconds, and a 115° reorientation field angle [6]. PFGE was carried out in 0.5 × TBE buffer chilled at 14°C for a total running time of 96 h. *Saccharomyces cervisiae *and *Schizosaccharomyces pombe *yeast chromosomes were used as DNA size markers (Bio-Rad). Gels were stained overnight with 0.5 μg/mL ethidium bromide, washed and transilluminated at 302 nm. Fluorescence intensity of the DNA was acquired, and transformed to arbitrary units of optical density using a digital imaging analysis system (GelDoc 2000 and Quantity One software, Bio-Rad). The sum of fluorescence within DNA smears was used for calculations and assessing differences in between experiments.

### Determination of H2AX and activated caspase-3 by immunofluorescence

Cryostat sections (3-μm thick) of cell-plugs embedded in Optimal Cutting Temperature OCT-compound (Sakura Finetek Europe, Alphen aan Den Rijn, The Netherlands) were used to determine histone H2AX phosphorylation (γH2AX) or activation of apoptosis by cleavage of caspase-3 [15,16]. Samples were fixed with 4% neutral-buffered formaldehyde, washed (0.1% triton in PBS for 10 min) and incubated for 1 hour with protein-blocking solution. Next, the slides were incubated with primary antibodies anti-phospho-histone H2AX (ser139) (Millipore-Upstate, Billerica, MA, US) or cleaved caspase-3 (Asp175) (Cell Signaling Technology, Danvers, MA, US) followed by incubation with secondary antibodies Alexa Fluor 488-conjugated and Alexa Fluor 594-conjugated (Invitrogen, Carlsbad, CA, US), respectively, all at a dilution of 1:500 for 1 h, at room temperature. Fluorescence images were captured by using a Zeiss Axioplan 2 imaging epi-fluorescence microscope equipped with a charge-coupled device camera and SPOT advanced software (Diagnostic Instruments Inc, Sterling Heights, MI, US). Five to ten randomly selected field microscopic images per slide were analyzed. Cells were counted using the ImageJ program, public domain Java image processing software (http://rsb.info.nih.gov/ij/).

Activation of apoptosis in cells growing in monolayer was examined using immunofluorescence by the specific nuclear TO-PRO-3 dye (Invitrogen), cleaved caspase-3 (Asp175), and Alexa Fluor 488-conjugated antibodies.

### Caspase inhibitors

To inhibit apoptosis, cell-plugs were treated with a pancaspase inhibitor, the halomethyl ketone z-vad-fmk (Bachem, Bubendorf, Switzerland). Cells obtained from xenografts were treated with the caspase-3 inhibitor zvd-fmk (Bachem) instead of z-vad-fmk substance. Both substances were incubated for 1 h before cell encapsulation and during incubation at 10 μM and 100 μM concentration, respectively.

### Statistics

Results were expressed as mean ± standard error (SE). Statistically significant differences in between-group comparisons were defined by using a two-tailed significance level of *P *< 0.05. The Statistical Package for Social Sciences, version 13.0 (IBM, Madrid, Spain) was used for data analysis.

## Results

**Figure 1:**
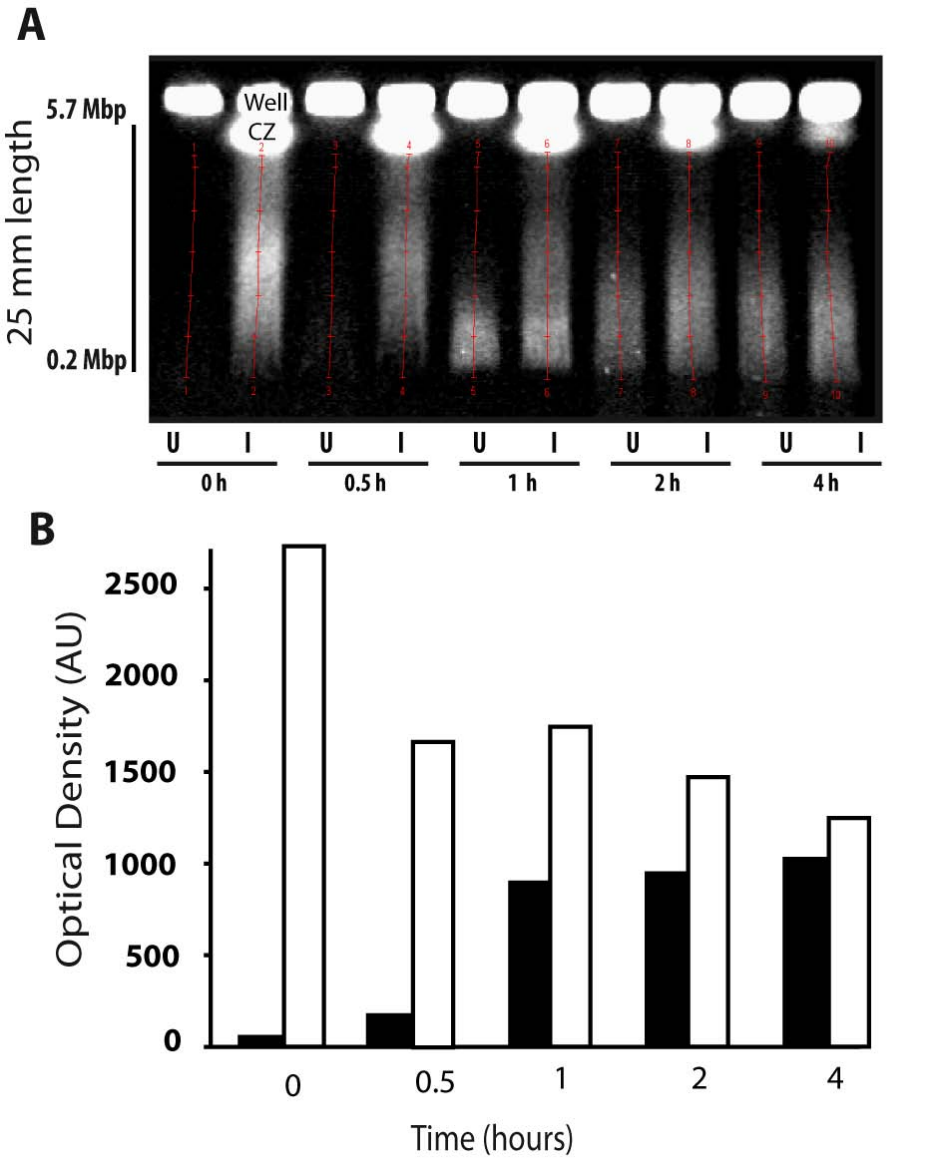
**PFGE pattern**. (A) PFGE for a typical A431 cell line experiment with agarose-encapsulated cells. The range of DNA quantification, beginning just before the compression zone (CZ) and ending at the bottom of the smears, is shown. (B) The amount of released DNA (optical density, Arbitrary Units, AU) was plotted as a function of time using the following time-points 0, 0.5, 1, 2 or 4 h. *U *stands for unirradiated (black bars) and *I *for 45 Gy irradiated cell-plugs (white bars). Data were obtained from one experiment.

Irrespectively whether cells were irradiated or not, we found that the pattern of DNA smears consisted of a compression zone (CZ) just below the wells, which was more patent in irradiated cells, followed with an area of distribution of DNA fragments ending in a sudden edge (Figure 1). The GelDoc settings for fluorescence acquisition were adjusted (exposure time and iris aperture) in each gel to obtain smears below saturation levels but sufficiently high to be measured. Thus, the range of fluorescence could vary depending on whether the gel contained unirradiated or irradiated cell plugs. In unirradiated cell-plugs, we observed an initial low extraction of DNA which progressively increased over time. In the A431 cell line, this spontaneous DNA fragmentation increased significantly over the incubation time (Figure 1), and similar figures were seen in the NP18 cell line (data not shown). In sharp contrast, in irradiated cell-plugs the initial DNA extraction was higher than in unirradiated cell-plugs indicating that radiation-induced DNA breakage occurred in a manner quantifiable by PFGE. Moreover, in this irradiated plugs we found a time-dependent decrease in DNA fragmentation compatible with the process of rejoining (Figure 1). Although some rejoining might have taken place in this experimental setting, proper estimation of DNA repair was clearly interfered by spontaneous DNA fragmentation, logically occurring in both unirradiated and irradiated cell-plugs.

**Figure 2:**
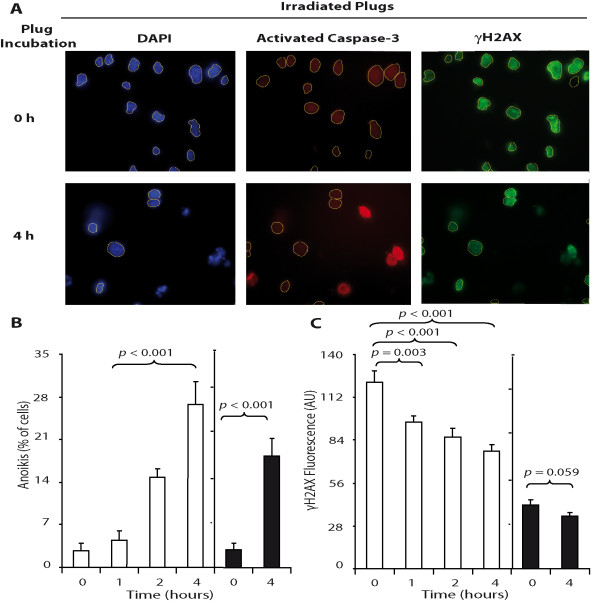
**Caspase-3 activation increased and radiation-induced γH2AX fluorescence decreased in a time-dependent manner**. (A) NP18 cell-plugs were irradiated with 45 Gy and incubated to allow for 0, 1, 2 or 4 h of repair. Illustrative DAPI stained nuclei (blue), fluorescence from caspase-activated cells (red), and γH2AX fluorescence (green) pictures are shown at 0 and 4 hours incubation times. Regions of interest (ROI) are depicted (original magnification, ×1000). (B) NP18 cell-plugs were irradiated (white bars) or not (black bars) and anoikis (% of cells showing activated caspase in 100 cells counted per time point) were determined. (C) In the same cells as in panel B, γH2X fluorescence (within DAPI-ROI in 150 nuclei counted per time point) was measured in caspase-negative cells. Data were obtained from 2 independent experiments. *P*-values were calculated using the Mann-Whitney test.

**Figure 3:**
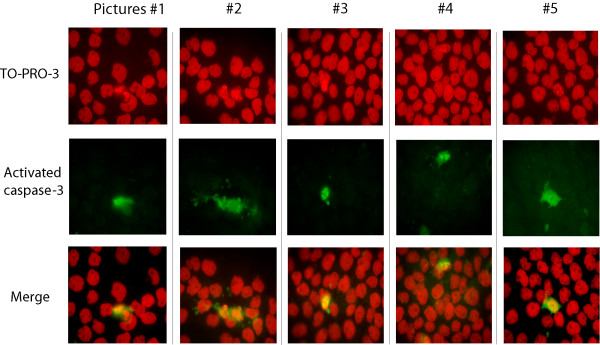
**Apoptosis in irradiated cells growing as monolayer was negligible**. NP18 cells growing on glass coverslip were irradiated with 45 Gy and incubated for 4 hours. Shown are five microscopic pictures (original magnification, ×1000): TO-PRO-3 stained nuclei (red), fluorescence from caspase-3-activated cells (green), and merged pictures. In sharp contrast with Figure 2B, apoptosis was negligible. Despite irradiation, the typical nuclear fragmentation and chromatin condensation associated with apoptosis was minimal, but consistent with the specific activation of caspase-3.

Given that agarose is a non-physiological environment, we hypothesized that spontaneous DNA degradation observed in PFGE could be a consequence of apoptosis triggered by agarose associated with the loss of normal cell-matrix interactions. To evaluate this possibility we examined apoptosis activation in cells embedded in agarose. Thus, we decided to determine cleavaged caspase-3 levels, an apoptosis effector, in cell-plugs (Figure 2A). Cleavaged caspase-3 was gradually activated regardless of whether the cells were irradiated or not, suggesting that apoptosis was triggered by agarose environment (Figure 2B). To further confirm that the origin of apoptosis was the encapsulation in agarose, we investigated the activation of caspase-3 in cells growing as monolayer (in a physiological environment). We observed only a minimal activation of apoptosis after 45 Gy (of 174 cells evaluated, 6 were caspase positive after 4 hours of incubation time: 3.44% of cell population), a finding that also precludes a substantial radiation-induced origin of apoptosis and corroborates the participation of agarose in spontaneous DNA fragmentation (Figure 3). To study whether apoptosis and repair could coexist we determined simultaneously the activation of caspase-3 and histone γH2AX by a double staining technique. Immediately following irradiation we observed high levels of γH2AX induction and low levels of cleavaged caspase-3 (Figure 2A). However, over incubation time we found that a fraction of apoptotic cells was simultaneously visible with a relevant fraction of cells in which the γH2AX was present (Figure 2A). As we were able to exclude caspase-positive cells from γH2AX fluorescence measurements we could specifically determine the evolution of DNA-DSBs. We found that initial radiation-induced γH2AX fluorescence decreased significantly over the incubation time, suggesting that in those cells in which apoptosis was not triggered radiation-induced damage could be repaired (Figure 2C). Interestingly, while the intensity of γH2AX decreased in irradiated cell plugs, the γH2AX levels in unirradiated plugs did not vary, indicating that γH2AX levels in those cells were not significantly influenced by agarose (Figure 2C).

**Figure 4:**
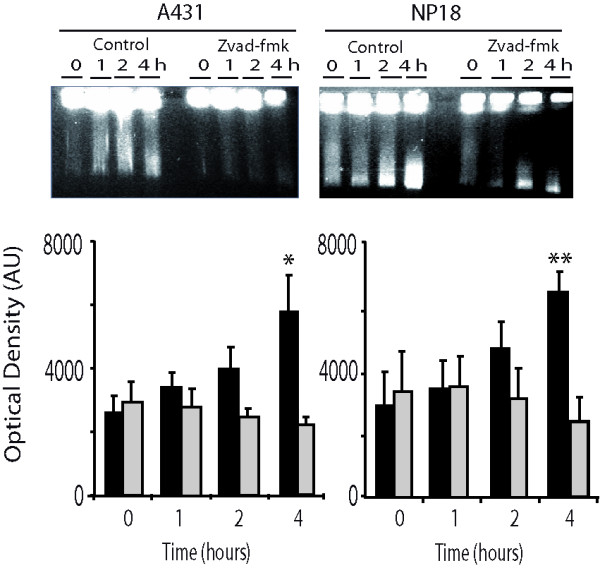
**Caspase inhibitors blocked apoptosis induced by agarose encapsulation**. Representative PFGE from A431 and NP18 cell lines are depicted. Spontaneous DNA fragmentation induced exclusively by incubation in unirradiated cell-plugs (black bars) was inhibited (grey bars) by zvad-fmk (100 μM). *P = 0.016; **P = 0.083 (Mann-Whitney test, 4 independent experiments). No significant differences were found between the A431 and N18 cell lines.

To further verify that the spontaneous DNA fragmentation observed in the PGFE experiments was induced by apoptosis, we treated cell-plugs with the pancaspase inhibitor z-vad-fmk. First, we corroborated that merely incubating cell-plugs was sufficient to induce DNA fragmentation in a time-dependent manner (Figure 4). We next found that treatment of these cell-plugs with the anti-apoptotic agent inhibited progressive spontaneous DNA degradation, confirming the crucial role of apoptosis and corroborating the mechanistic explanation of the caspase-3 activation we demonstrated by immunofluorescence method in cell-plugs (Figure 4).

**Figure 5:**
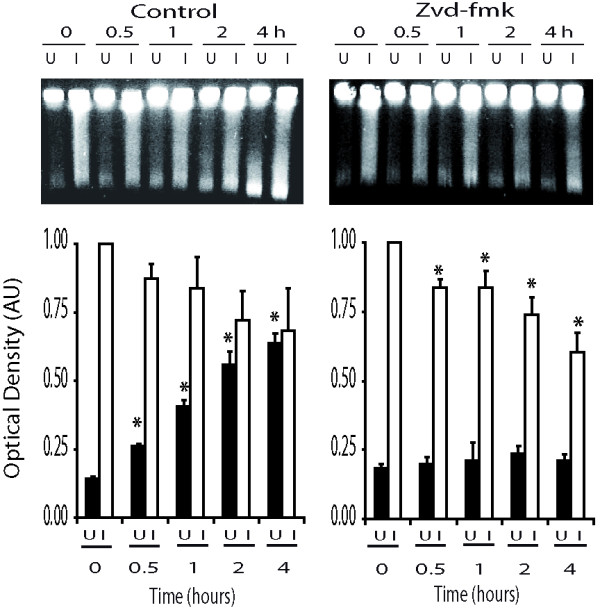
**Reduction of apoptosis improved the estimation of DNA repair in NP18 xenografts**. PFGE was performed in unirradiated (black bars) and irradiated (45 Gy; white bars) cell-plugs obtained from xenografts (NP18) incubated in absence or presence of zvd-fmk (10 μM). Optical density was normalized to radiation-induced initial damage in absence of, or presence of zvd-fmk. In absence of zvd-fmk, spontaneous DNA fragmentation (black bars) increased significantly while radiation-induced breakage (white bars) decreased over time-course. In presence of zvd-fmk, radiation-induced fragmentation decreased but spontaneous DNA breakage was blocked. *P < 0.05 compared to optical density at time 0 h (Mann-Whitney test, 4 independent experiments).

Since encapsulating cells before irradiation is particularly useful in a clinical setting with tumour biopsies, our aim was to investigate whether the inhibition of apoptosis would facilitate the evaluation DNA repair in this context. To do this, we removed tumours (< 1000 mm^3^) derived from NP18 cells from the subcutaneous tissue of nude mice. In plugs containing cells from xenografts we reproduced the DNA fragmentation pattern previously observed in PFGE of cultured cells (shown in Figure 1). In irradiated cell-plugs, radiation-induced DNA-DSB decreased progressively, whereas spontaneous fragmentation increased gradually (Figure 5). However, when cell-plugs were treated with zvd-fmk, a specific caspase-3 inhibitor, spontaneous DNA degradation was inhibited over the entire time-course (*P *> 0.3). Importantly, radiation induced DNA breakage, which is attributable to rejoining, continued diminishing (Figure 5). The blockade of apoptosis thus allowed us to efficiently determine rejoining without the inference of the apoptosis. Figure 5 illustrates the effect of abrogating apoptosis in NP18 cells from a xenograft, showing clear differences between initial and residual radiation-induced damage depending on the absence or presence of zvd-fmk. By inhibiting apoptosis, we were able to reduce spontaneous DNA breakage and estimate rejoining as the initial DNA released minus residual DNA released without the interference of apoptosis. On the other hand, the intensity of apoptosis could be easily estimated by subtracting the total DNA released in unirradiated zvd-treated cell-plugs from the unirradiated non-zvd-treated cell-plugs.

## Discussion

It is believed that specific tumour radiosensitivity is highly dependent on DNA repair and, reasonably, tumour response is also influenced by this biologic variable. Initially, PFGE was devised as a test for predicting cancer response to radiation therapy. However, soon thereafter the few studies that examined the utility of the PFGE method in tumour biopsies concluded that PFGE was insufficiently robust to be a predictive test, mainly due to spontaneous DNA fragmentation that occurs during sample processing. In the present study, we revisited the PFGE method to demonstrate that apoptosis induced by agarose is a component of total DNA breakage, and that the inhibition of apoptosis allows for more proper estimation of radiation-induced DNA DSB repair and apoptosis itself. First, we found spontaneous cleavage of caspase-3 in the cell-plugs, suggesting that the agarose environment triggered apoptosis activation, and second we inhibited spontaneous DNA fragmentation in PFGE by means of selective and specific caspase inhibitors. This is the first study to describe that the spontaneously released DNA in cell-plugs reflects an ongoing homeless-induced apoptosis, referred to as anoikis [17], when cells are embedded in agarose. DNA cleavage into large fragments is an early event observed in the apoptotic cascade before the typical endonuclease cleavage into 180 to 200 bp can be detected as a DNA ladder [18]. This DNA breakage is exactly what we observed in our experiments, and further supports the apoptotic aetiology of spontaneous degradation. Furthermore, the fact that we did not detect caspase-3 activation in irradiated cells growing as a monolayer, and caspase-3 activation was seen only in cells embedded in agarose (whether irradiated or not), allows us to rule out a radiation-induced origin of apoptosis.

After elucidating the mechanism behind DNA degradation, it seemed reasonable to conclude that apoptosis initiation was occurring in both unirradiated and irradiated cell-plugs. However, our findings show that γH2AX fluorescence, a surrogate marker of DNA-DSB [19], continued to decrease progressively, a result compatible with radiation-induced DNA-DSB repair. We should keep in mind that during incubation, apoptosis activation was triggered in a percentage of cells, whereas in non-apoptotic cells repair took place, as we were able to show by eliminating apoptotic cells from the quantification of fluorescence in cell-plugs. Thus, in our PFGE we obtained smears that represent the final stage of two independent processes--DNA repair and anoikis--at each time point.

This type of apoptosis is physiologically induced to prevent proliferation of cells at inappropriate locations. Tumour cells that lose this homeostatic control can manage to thrive in an anchorage-independent manner, an aggressive characteristic of different types of human malignancies. The clinical significance of resistance to anoikis is increasingly associated with malignant phenotypes, therapy resistance, and poor prognosis [20].

Finally, by incubating cell plugs in absence or presence of caspase inhibitors we were able to determine two traits associated with tumour aggressiveness, DNA repair and apoptosis. Reasonably, in a short time-course scheme, a relatively low DNA release rate will indicate an aggressive phenotype because the majority of radiation-induced DNA fragments are quickly rejoined (resistance to radiation) and apoptosis is not triggered (resistance to apoptosis). In this scenario, cancer cells would survive in an adverse environment (i.e., loss of extracellular matrix due to radiation cell killing) and would rapidly recover their proliferative potential. On the other hand, a significant smear would indicate a less aggressive behaviour due to low radiation repair and high sensitivity to anoikis.

## Conclusions

Our study confirms previous findings that apoptosis occurring in agarose-encapsulated cells interferes with PFGE evaluation of radiation-induced DNA repair analysis. However, we found that it is possible to reduce this interference by using caspase inhibitors, thereby greatly improving the estimation of DNA repair in tumour cells. Simultaneously, apoptosis induced by agarose can also be determined. The ability to determine together two traits--repair and apoptosis--involved in cell fate opens new possibilities for PFGE as functional assay.

## Competing interests

The authors declare that they have no competing interests.

## Authors' contributions

JB conceived the study and drafted the manuscript. GP, LL and MB participated in PFGE and in immunofluorescence studies. XS provided informatics and support with statistics for data analysis. SM, OC, RM and GC participated importantly in the conception, design of the study and helped to draft the manuscript. All authors read and approved the final manuscript.
